# Differences According to Sex in Short-term Clinical Outcomes After Supraventricular Tachycardia Catheter Ablation: Insights from the Nationwide Readmission Database

**DOI:** 10.19102/icrm.2022.13105

**Published:** 2022-10-15

**Authors:** Mahmoud Khalil, Ahmed Maraey, Muhammad Haisum Maqsood, Ahmed M. Elzanaty, Mahmoud Salem, Ahmed Younes, Hadeer Elsharnoby, Kenneth Ong, Mohamed Shokr

**Affiliations:** ^1^Internal Medicine Department, Lincoln Medical and Mental Health Center, Bronx, NY, USA; ^2^Cardiovascular Medicine Department, Tanta University, Tanta, Egypt; ^3^Department of Internal Medicine, CHI St. Alexius Health/University of North Dakota, Bismarck, ND, USA; ^4^Cardiovascular Medicine Department, University of Toledo, Toledo, OH, USA; ^5^Center for Advanced Heart and Lung Diseases, Baylor University Medical Center, Dallas, TX, USA; ^6^Internal Medicine Department, East Carolina University, Greenville, NC, USA; ^7^Cardiovascular Medicine Department, Lincoln Medical and Mental Health Center, New York, NY, USA; ^8^Leon H. Charney Division of Cardiology, Cardiac Electrophysiology, NYU Langone Health, New York University School of Medicine, New York, NY, USA

**Keywords:** Ablation, gender differences, national readmission database, supraventricular tachycardia

## Abstract

Catheter ablation is indicated for the management of supraventricular tachycardias (SVTs). We investigated the effect of sex on short-term outcomes after catheter ablation for SVTs other than atrial fibrillation (AF). Using the Healthcare Cost and Utilization Project Nationwide Readmission Database for the years 2016–2018, SVT patients who underwent catheter ablation were identified using the appropriate International Classification of Diseases, 10th Revision, codes. The primary outcome was 30-day readmissions. Secondary outcomes included 30-day readmissions for SVT, postprocedural bleeding, acute myocardial infarction, transient ischemic attack, stroke, hemopericardium, cardiac tamponade, length of stay (LOS) in the hospital, and total hospital charges (in USD). Female sex was not associated with an increased risk of the primary outcome (*P* = .168) but was associated with a significantly decreased risk of postprocedural revascularization (*P* = .001), LOS (*P* = .003), and total hospital charges (*P* = .002). There were no significant differences in other secondary outcomes. Among patients admitted for catheter ablation for SVTs (other than AF), female sex was associated with decreased LOS and total hospital charges, which may be attributed to increased comorbidity rates in men and gender-based biases.

## Introduction

Supraventricular tachycardia (SVT) is defined as an abnormal rhythm that originates from the His-bundle tissue or above. Catheter ablation is class I and level of evidence B non-randomized for symptomatic focal atrial tachycardia, atrioventricular (AV) nodal re-entrant tachycardia (ablation of slow pathway), orthodromic AV re-entrant tachycardia (ablation of accessory pathway), and cavotricuspid isthmus ablation in atrial flutter.^1^ Atrial fibrillation (AF) catheter ablation has shown sex dissimilarities in adverse outcomes.^2–5^ However, there are no studies on sex-based catheter ablation outcomes in SVTs excluding AF. We hypothesized that, due to gender disparities and delays in medical treatment, female patients undergoing SVT ablation would have different outcomes than male patients. We sought to analyze sex differences in short-term adverse outcomes of catheter ablation in patients with SVTs excluding AF.

## Methods

### Data source

This is a retrospective cohort study using the Agency for Healthcare Research and Quality’s Healthcare Cost and Utilization Project (HCUP) Nationwide Readmission Database (NRD) for the years 2016–2018. The NRD is the largest publicly available all-payer inpatient health care readmission database in the United States. The NRD is drawn from HCUP State Inpatient Databases containing verified patient linkage numbers that can be used to track a person across hospitals within a state, while adhering to strict privacy guidelines.

The NRD contains both patient- and hospital-level information. Up to 40 discharge diagnoses and 25 procedures are collected for each patient using the International Classification of Diseases, 10th Revision, Clinical Modification (ICD-10-CM). National estimates were produced using sampling weights provided by the sponsor (HCUP). All values presented are weighted estimates.

### Study population

Our study population comprised patients admitted with a primary diagnosis of SVT who underwent catheter ablation (ICD-10 codes 02563ZZ, 02573ZZ, 02553ZZ, and 02583ZZ). Patients were excluded if they were <18 years of age; had a secondary diagnosis of ventricular tachycardia, AF, ventricular premature beats, or unspecified paroxysmal tachycardia; or had missing data. Also, to avoid inclusion of patients undergoing ablation of the AV junction, we excluded patients with diagnostic or procedural codes indicating prior implantation of a pacemaker. December discharges and patients who died during the index admission were excluded when evaluating 30-day readmission outcomes. Patients who had catheter ablation were identified using ICD-10 codes (02563ZZ, 02573ZZ, 02553ZZ, and 02583ZZ). NRD variables were used to identify patients and hospital characteristics. Patient characteristics included age, sex, median household income, and primary insurance. Hospital characteristics included hospital bed size and teaching status.

In accordance with the HCUP data use agreement, we excluded any variable containing a small number of observations (≤10) that could pose a risk of identification of the person or data privacy violation.

### Study outcomes

The primary outcome was 30-day readmission after index admission. Index admission was defined as the first admission with a diagnosis of SVT without prior admission in a period of 30 days. A readmission was defined as any readmission within 30 days of the index admission. If the patient was readmitted multiple times during the 30 days post-admission, only the first readmission was included.

Secondary outcomes were length of stay (LOS) in the hospital, total charges (in USD), iatrogenic cardiac complications, postprocedural bleeding, cardiac tamponade, hemopericardium, stroke and transient ischemic attack (TIA), and readmission with a primary diagnosis of SVT within 30 days. LOS and total hospital charges were directly coded in the NRD.

### Statistical analysis

Data analysis was performed using STATA 17 (StataCorp, LLC, College Station, TX, USA). Continuous variables were compared using Student’s *t* test, and categorical variables were compared using the chi-squared test. Univariate regression analysis was used to calculate unadjusted odds ratios (ORs) for the primary and secondary outcomes. A multivariate regression analysis was used to adjust for confounders and calculate adjusted ORs (aORs). Confounders were adjusted for age; median income; primary insurance; hospital bed size; hospital teaching status; and chronic comorbidities such as hypertension, diabetes mellitus, congestive heart failure, valvular heart disease, peripheral vascular disease, chronic lung disease, obesity, liver disease, history of myocardial infarction (MI), history of percutaneous coronary intervention (PCI), and history of coronary artery bypass grafting (CABG). Logistic regression was used for dichotomous outcomes, and linear regression was used for continuous outcomes. All *P* values were 2-sided, with .05 used as the threshold for statistical significance.

## Results

From 107 million discharges included in the NRD from 2016–2018, we identified a total of 14,724 patients who presented with SVT and underwent catheter ablation. Baseline characteristics were stratified according to sex.

The mean age was 59 (± 15) years in men and 58 (± 16) years in women. Women were less likely to have a Carlson comorbidity score of ≥3 points (17% vs. 25%, *P* < .001), congestive heart failure (21% vs. 31%, *P* < .001), a history of MI (5% vs. 10%, *P* < .001), a history of PCI (5% vs. 11%, *P* < .001), or a history of CABG (2% vs. 8%, *P* < .001). Other patient and hospital characteristics were not different between the 2 groups **(Table 1)**.

Of the 14,724 patients who survived the index admission, 1,376 patients were readmitted within 30 days. Six hundred eighty-five (9%) were women and 691 (10%) were men. Female sex was not associated with an increased risk of 30-day all-cause readmission (aOR, 0.99; 95% confidence interval [CI], 0.83–1.17; *P* = .93).

The most common primary etiology in both sexes was SVT recurrence (11.5% in women and 12.9% in men). The second most common etiologies were pulmonary embolism in women (4.5%) and sepsis in men (6.4%), respectively.

A total of 148 readmissions (73 [1%] women vs. 75 [1%] men) carried a primary diagnosis of SVT. Female sex was not associated with an increased risk of 30-day SVT readmission (aOR, 0.84; 95% CI, 0.44–1.33; *P* = .47).

A total of 278 patients (76 [1%] women vs. 202 [3%] men) required coronary revascularization during the index admission. Female sex was associated with decreased odds of acute MI (aOR, 0.38; 95% CI, 0.2–0.6; *P* < .01).

A total of 682 patients (351 [5%] women vs. 331 [5%] men) had advanced heart block (second- or third-degree AV block). Temporary pacemaker implantation was performed in 117 patients, including 70 (1%) women and 47 (1%) men. No sex difference existed in the rate of heart block (aOR, 1.05; 95% CI, 0.83–1.33; *P* = .644) or temporary pacemaker implantation (aOR, 1.54; 95% CI, 0.9–2.62; *P* = .12).

Iatrogenic cardiac complications and postprocedural hemorrhage occurred in 150 patients (84 [1%] women vs. 66 [1%] men) and 71 patients (27 [<1%] women vs. 44 [<1%] men), respectively. Female sex was not associated with an increased odds of iatrogenic cardiac complications (aOR, 1.11; 95% CI, 0.68–1.8; *P* = .681) or postprocedural hemorrhage (aOR, 0.56; 95% CI, 0.28–1.13; *P* = .108).

Stroke or TIA occurred in 117 patients, including 48 (1%) women and 69 (1%) men, during the index admission. Similar to previously mentioned outcomes, no sex difference was observed (aOR, 0.75; 95% CI, 0.43–1.31; *P* = .31).

The mean LOS in our cohort was 4.5 days. Notably, the mean LOS was different between the sexes (4.9 days in men vs. 4.1 days in women, *P* < .001). After adjustment, female sex was associated with a decreased mean LOS of 0.49 days, and that difference was statistically significant (*P* = .001).

**Figure 1:**
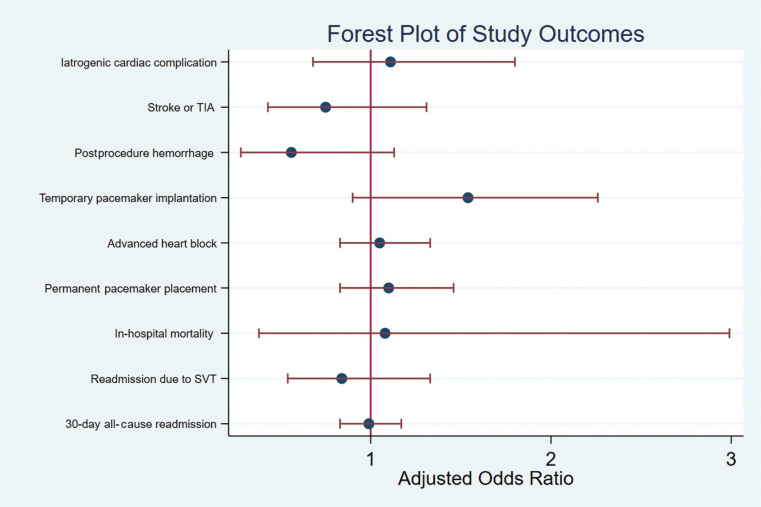
Forest plot for the study outcomes. *Abbreviations:* CI, confidence interval; SVT, supraventricular tachycardia; TIA, transient ischemic attack.

The mean hospital charge amount in our total cohort was $129,134, and the mean total charge amount was different between women ($122,086) and men ($136,627) (*P* < .001). After adjustment, female sex was associated with a reduced mean total hospital charge amount of $10,459 compared to men, and that difference was statistically significant (*P* < .001) **(Table 2, Figure 1)**.

## Discussion

This nationally representative analysis of 14,724 SVT patients who underwent ablation during hospital admission, using NRD data from 2016–2018, found a non-significant difference in primary (30-day readmission) and secondary outcomes (including in-hospital mortality, postprocedural hemorrhage, iatrogenic cardiac complications, advanced heart block, temporary pacemaker implantation, stroke/TIA, and readmission for SVT). However, there was a significantly higher comorbidity burden and a greater probability of revascularization, an increased mean LOS, and a higher hospital charge amount in men compared to women.

Available evidence suggesting sex-based outcomes of radiofrequency ablation largely stems from studies of patients with AF.^2–5^ For example, the 1STOP project, a study of 2,125 patients from 47 cardiology centers in Italy, showed non-significant differences in procedure-related complications between the 2 sexes.^4^ However, the study cohort was only 27% female. Another NRD analysis conducted from 2010–2014 on AF patients revealed higher ablation-related complication and 30-day readmission rates in women but lower cumulative costs.^5^ Similarly, Kaiser et al. found greater hospitalization rates in women with lower rates of cardioversion and repeat ablation.^2^ Our study, which had a slightly larger proportion of women compared to prior studies, demonstrated non-significant differences in outcomes with lower cumulative costs and a shorter LOS for women, which may indicate improvements in overall care. The differences in LOS and costs could also be attributed to greater rates of revascularization and a higher comorbidity burden in men compared to women. Further, reductions in cost and LOS in women could also stem from a gender bias—women are more likely to have their cardiac symptoms ignored by physicians,^6^ experience a delay in medical procedures,^6,7^ and fail more anti-arrhythmic medications before proceeding to ablation.^8,9^ Similarly, women are also more likely to be misdiagnosed for paroxysmal SVT.^10^ The aforementioned gender bias can have a role in delayed diagnoses of comorbid conditions and subsequent lower rates of MI.

Patients with coronary artery disease are at a higher risk for AF and recurrent AF. There is decreased recurrence of AF in patients who undergo coronary artery revascularization.^11,12^ There is a paucity of literature on revascularization and SVT burden in patients with SVTs excluding AF. In the present study, we found higher revascularization procedures in men compared to women. It would be interesting to see results of future studies of the impact of revascularization on SVT burden.

Though a rare entity in the literature, postablation coronary injury was more common in men than women in our study. This may be explained by increased baseline comorbidities in the male group, including a history of MI, CABG, PCI, and congestive heart failure. The possible mechanism of MI after ablation is thought to be due to radiofrequency-associated damage to coronary arteries from anatomical variations.^13,14^ This merits further studies on the mechanism of post-ablation MI, systems and processes to mitigate the risks as well as effective treatment to avoid catastrophic outcomes.

This study should be interpreted considering some important limitations. First, it is an observational study, and the contribution of confounding factors on overall outcomes is unknown. Second, clinical and procedural characteristics, such as ejection fraction, body mass index, and type of ablation, were not available. Third, the real-world, all-payer, nationally representative NRD cohort does not include all admissions in the United States, so this study might over- or underestimate the overall difference in outcomes. Also, outpatient procedures, which mainly constitute the majority of SVT ablation procedures, could not be tracked in this database.

## Conclusion

Compared to men, women who underwent catheter ablation of SVT did not have a statistically significant difference in 30-day readmission or procedural complications such as heart block, hemorrhage, or stroke. Women had a shorter hospital stay and less hospital costs than men, and men had more baseline comorbidities at the ablation time. More research is needed to further explore the reasons for such differences.

## Figures and Tables

**Table 1:** Baseline Characteristics of the Study Population

Characteristics	SVT Ablation (n = 14,724)		*P* Value
	Male (n = 7,140)	Female (n = 7,584)	
**Mean age in years (SD)**	59 (±15)	58 (±16)	.048
**Smoking**	1,264 (18%)	944 (12%)	<.001
**Charlson comorbidity index score**			<.001
0	2,240 (31%)	3,177 (42%)	
1	1,748 (24%)	1,941 (26%)	
2	1,225 (17%)	1,044 (14%)	
≥3	1,927 (27%)	1,422 (19%)	
**Median household income quartile for zip code (USD)**			.012
$1–$45,999	1,925 (27%)	2,281 (31%)	
$46,000–$58,999	1,913 (27%)	1,829 (24%)	
$59,000–$78,999	1,645 (23%)	1,721 (23%)	
≥$79,000	1,574 (22%)	1,641 (22%)	
**Insurance**			
Medicare	3,215 (45%)	3,555 (48%)	<.001
Medicaid	921 (13%)	1,094 (14%)	
Private	2,405 (34%)	2,518(33%)	
Self-pay	274 (4%)	182 (2%)	
No charge	61 (1%)	53 (1%)	
Other	255 (4%)	167 (2%)	
**Hospital bed size**			.38
Small	468 (7%)	481 (6%)	
Medium	1,662 (23%)	1,882 (25%)	
Large	5,010 (70%)	5,220 (69%)	
**Teaching hospital**	6,186 (87%)	6,530 (86%)	.54
**Comorbidities**			
Hypertension	4,702 (66%)	4,535 (60%)	<.001
Chronic kidney disease	1,275 (18%)	819 (11%)	<.001
History of MI	689 (10%)	411 (5%)	<.001
History of PCI	755 (11%)	379 (5%)	<.001
History of CABG	581 (8%)	177 (2%)	<.001
Congestive heart failure	2,215 (31%)	1,564 (21%)	<.001
Valvular heart disease	934 (13%)	1,002 (13%)	.87
Peripheral vascular disease	680 (10%)	503 (7%)	<.001
Pulmonary circulatory disturbance	393 (6%)	457 (6%)	.33
Chronic lung disease	1,282 (18%)	1,573 (21%)	.003
Obesity	1,337 (19%)	1,500 (20%)	.27
Hypothyroidism	475 (7%)	1,285 (17%)	<.001
Diabetes mellitus	1,961 (13%)	1,802 (10%)	<.001
Liver disease	327 (5%)	199 (3%)	<.001
Alcohol abuse	552 (8%)	176 (2%)	<.001
Drug abuse	400 (6%)	194 (3%)	<.001

*Abbreviations:* CABG, coronary artery bypass grafting; MI, myocardial infarction; PCI, percutaneous coronary intervention; SD, standard deviation; SVT, supraventricular tachycardia.

**Table 2:** Outcomes of the Study

Outcome	SVT Ablation (n = 14,724)		*P* Value
	Male (n = 7,140)	Female (n = 7,584)	
**30-day readmission**	691 (10%)	685 (9%)	.34
Adjusted odds ratio (95% CI)	Reference	0.99 (0.83–1.17)	.93
**Readmission due to SVT**	75 (1%)	73 (1%)	.68
Adjusted odds ratio (95% CI)	Reference	0.84 (0.54–1.33)	.47
***In-hospital mortality (n)***	18 (<1%)	16 (<1%)	.66
Adjusted odds ratio (95% CI)	Reference	1.08 (0.38–2.99)	.89
**Permanent pacemaker placement**	240 (3%)	298 (4%)	.21
Adjusted odds ratio (95% CI)		1.10 (0.83–1.46)	.50
***Need for revascularization***	202 (3%)	76 (1%)	<.01
Adjusted odds ratio (95% CI)	Reference	0.38 (0.2–0.6)	.001
**Advanced heart block**	331 (5%)	351 (5%)	.99
Adjusted odds ratio (95% CI)	Reference	1.05 (0.83–1.33)	.644
**Temporary pacemaker implantation**	47 (1%)	70 (1%)	.25
Adjusted odds ratio (95% CI)	Reference	1.54 (0.9–2.62)	.12
**Postprocedural hemorrhage**	44 (1%)	27 (<1%)	.1
Adjusted odds ratio (95% CI)	Reference	0.56 (0.28–1.13)	.108
**Stroke or TIA**	69 (1%)	48 (1%)	.12
Adjusted odds ratio (95% CI)	Reference	0.75 (0.43–1.31)	.31
**Iatrogenic cardiac complication**	66 (1%)	84 (1%)	.47
Adjusted odds ratio (95% CI)	Reference	1.11 (0.68–1.8)	.68
**Mean length of stay (days)**	4.9	4.1	<.001
Adjusted mean difference (β) in days	Reference	−0.49 (−0.79, −0.20)	.001
**Mean total charges (USD)**	136,627	122,086	<.001
Adjusted mean total charges difference (β) in USD	Reference	−10,459 (−15,734, −5,138)	<.001

*Abbreviations:* CI, confidence interval; SVT, supraventricular tachycardia; TIA, transient ischemic attack.
